# Eight years of homicide evolution in Monterrey, Mexico: a network approach

**DOI:** 10.1038/s41598-020-78352-9

**Published:** 2020-12-09

**Authors:** Rodrigo Dorantes-Gilardi, Diana García-Cortés, Hiram Hernández-Ramos, Jesús Espinal-Enríquez

**Affiliations:** 1 Computational Genomics Division, National Institute of Genomic Medicine (INMEGEN), Mexico City, 14610 Mexico; 2 El Colegio de México (COLMEX), Mexico City, Mexico; 3 Facultad de Ciencias, Universidad Nacional Autónoma de México (UNAM), Mexico City, Mexico; 4 Centro de Ciencias de la Complejidad, Universidad Nacional Autónoma de México (UNAM), Mexico City, 04510 Mexico

**Keywords:** Applied physics, Complex networks, Nonlinear phenomena

## Abstract

Homicide is without doubt one of Mexico’s most important security problems, with data showing that this dismal kind of violence sky-rocketed shortly after the war on drugs was declared in 2007. Since then, violent war-like zones have appeared and disappeared throughout Mexico, causing unfathomable human, social and economic losses. One of the most emblematic of these zones is the Monterrey metropolitan area (MMA), a central scenario in the narco-war. Being an important metropolitan area in Mexico and a business hub, MMA has counted hundreds to thousands of casualties. In spite of several approaches being developed to understand and analyze crime in general, and homicide in particular, the lack of accurate spatio-temporal homicide data results in incomplete descriptions. In order to describe the manner in which violence has evolved and spread in time and space through the city, here we propose a network-based approach. For this purpose, we define a homicide network where nodes are geographical entities that are connected through spatial and temporal relationships. We analyzed the time series of homicides in different municipalities and neighborhoods of the MMA, to observe whether or not a global correlation appeared. We studied the spatial correlation between neighborhoods where homicides took place, to observe whether distance is a factor of influence in the frequency of homicides. We constructed yearly co-occurrence networks, by correlating neighborhoods with homicides happening within a same week, and counting the co-occurrences of these neighborhood pairs in 1 year. We also constructed a crime network by aggregating all data of homicides, eliminating the temporal correlation, in order to observe whether homicide clusters appeared, and what those clusters were distributed geographically. Finally, we correlated the location and frequency of homicides with roads, freeways and highways, to observe if a trend in the homicidal violence appeared. Our network approach in the homicide evolution of MMA allows us to identify that (1) analyzing the whole 86-month period, we observed a correlation between close cities, which decreases in distant places. (2) at neighborhood level, correlations are not distance-dependent, on the contrary, highest co-occurrences appeared between distant neighborhoods and a polygon formed by close neighborhoods in downtown Monterrey. Moreover, (3) An elevated number of homicides occur close to the 85th freeway, which connects MMA with the US border. (4) Some socioeconomic barriers determine the presence of homicide violence. Finally, (5) we show a relation between homicidal crime and the urban landscape by studying the distance of safe and violent neighborhoods to the closest highway and by studying the evolution of highway and crime distance over the cartel-related years and the following period. With this approach, we are able to describe the spatial and temporal evolution of homicidal crime in a metropolitan area.

## Introduction

### Violence in Mexico during the drug war

In an attempt to legitimize his questionable victory on the 2006 presidential elections, former Mexican president, Felipe Calderon Hinojosa (FCH), launched an intervention of the Mexican Army and Federal Police into the State of Michoacán, to face the drug cartels that operated in that region^1^. “Operativo Conjunto Michoacán” was the starting point of FCH’s Drug War^2^. After that, FCH launched other operations in the states of Guerrero, Baja California, Sinaloa, Nuevo León, Tamaulipas, Durango and Chihuahua^3^. This battle between Mexican security forces and drug cartels spread the violence throughout the whole country and homicide rates scaled to levels never seen before in Mexico^4^.

The dramatic increase of drug-related homicides during FCH’s drug war, brought a wave of violence as a collateral effect to the aforementioned war. Constant and documented human rights violations from security forces and the Mexican Army, disappearances, and displacement of thousands of people also incremented during FCH’s drug war^5^. In the first 3 years of the administration of the next president, Enrique Peña Nieto (EPN), there was an apparent period of lower homicide rates. However, at the beginning of 2015 a renewed wave of violence emerged at a national level and has not decreased since^6^.

**Figure 1:**
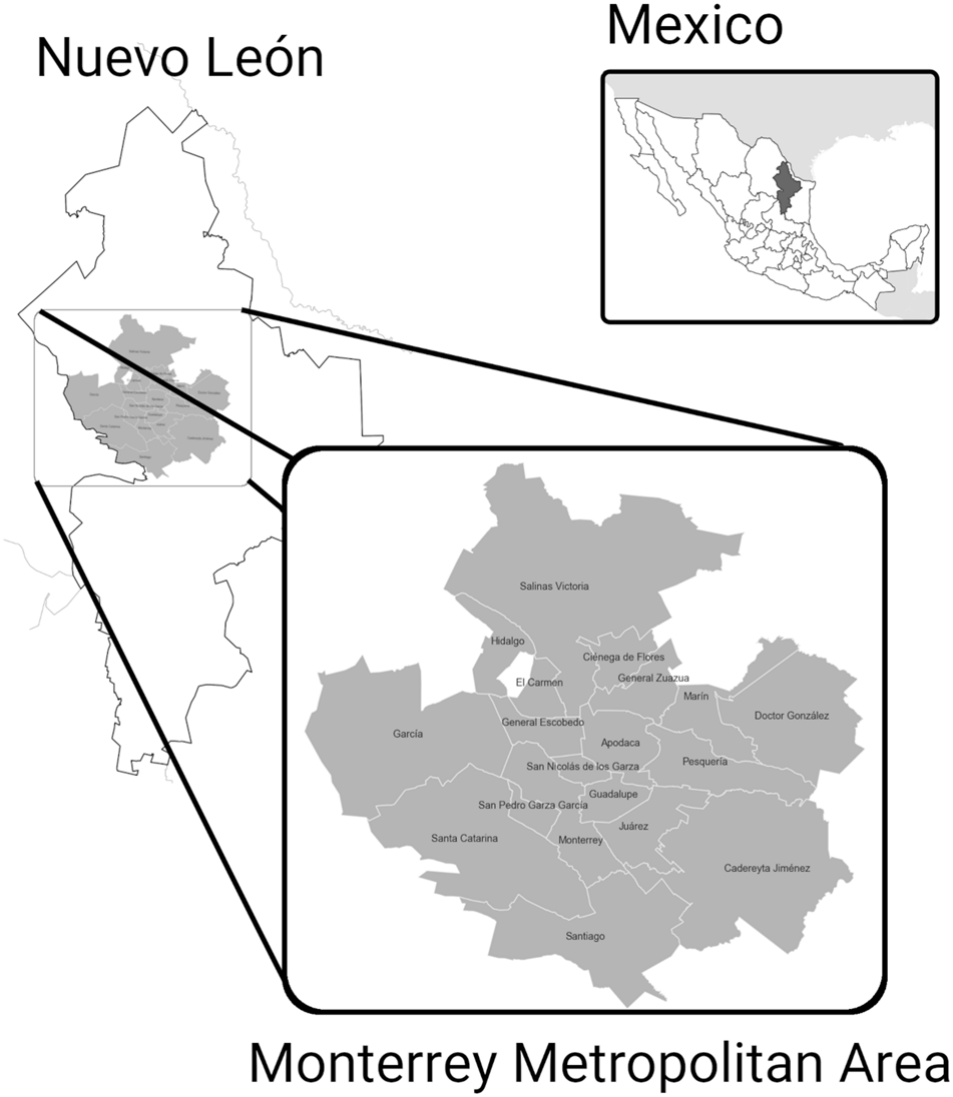
Political division of MMA. This map contains the names of municipalities in the MMA and their location inside the state of Nuevo León. It also displays where is Nuevo León located in Mexico.

FCH’s drug war also generated battles between drug cartels to control broad territories. In the year of 2009, there was a schism between the “Cartel del Golfo”, one of the most important criminal groups in Mexico, and the “Zetas” band, the former armed force of that cartel. This struggle took place in the northern states of Tamaulipas and Nuevo León. The violence triggered by this separation caused the highest homicide rates in the history of several cities in the region such as Monterrey^4^, and, more generally, in the Monterrey Metropolitan Area (MMA), the second largest urban area in Mexico, and an important industrial hub (Fig. 1).

In its darkest time, the *Zetas-Golfo* battle caused more that two hundred drug-related homicides just in one month in MMA^4^. Notwithstanding, the dramatic rise of homicidal crimes in MMA was also diminished during EPN’s administration, in particular during the Nuevo León administration of former Governor Rodrigo Medina. However, MMA has never returned to the low homicide levels previous to FCH’s incumbency.

As mentioned above, Felipe Calderón’s drug war caused an impressive increment in the homicidal violence in almost the whole surface of the country. However, the dynamics of that violence was not the same in all Mexican states. The local gang-related struggles mostly delineated the evolution of homicides.

**Figure 2:**
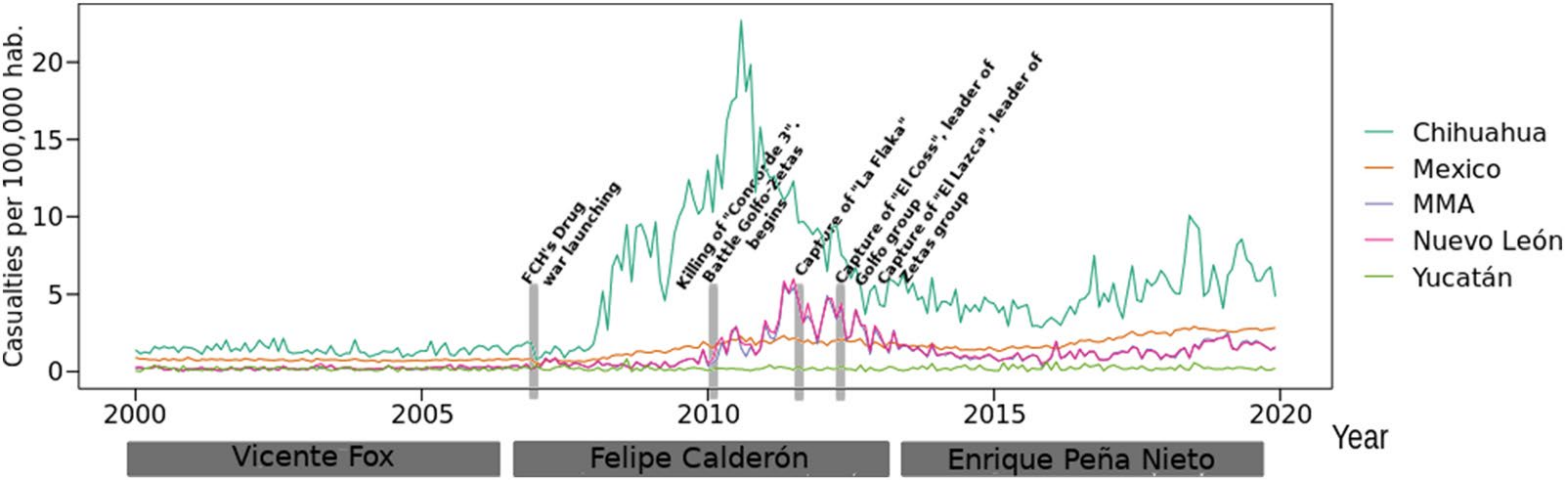
Timeseries of homicides in Mexico, Nuevo León State, and the Monterrey Metropolitan Area (MMA). In this plot, the monthly number of homicides for those three levels of granularity are described. It can be observed at the beginning of the FCH’s presidency, the substantial increase in homicide rate in the three levels. The second increase in homicides in Mexico did not follow the same trend in Nuevo León nor MMA, however, in both places violence increased. For comparison, Chihuahua and Yucatán states timeseries are also depicted. Grey bars indicate relevant moments in the drug war.

As an example of the latter, Fig. 2 shows the evolution of Mexico’s monthly homicides, Nuevo León State homicides, and also the MMA monthly casualties. As it can be clearly observed, the rise of violence in Mexico coincides with the arrival of FCH to the presidency and his **“guerra contra las drogas”**; however, Nuevo León State and its MMA presented a more dramatic increment in 2009–2010, but also a strong and constant decrease during the period 2012–2015.

Other Mexican states had a quite different behavior of homicide evolution. In the figure it is also shown the dynamics of homicide violence in Chihuahua and Yucatán states, the most and less violent places in México during the FCH’s war on drugs. Chihuahua had the highest rates of drug-related homicides. In 2010, Juárez, the most important border city of Chihuahua State, had almost 3,000 drug war-related casualties, 10 times more than those in 2008.

On the other hand, Yucatán, a Southeastern State, had one of the lowest homicide rates in that period. This shows how different the war on drugs in Mexico took place, depending on the territory and the criminal groups that had influence in those places. It is worth noticing that Yucatán State also had the presence of criminal groups. In fact Los Zetas group controlled the southernmost part of México during several years^7^.

A pertinent question raised by the observed differences between Mexican States in homicides, is why two States (Nuevo León and Yucatán) with the presence of the same group (Zetas) presented so dissimilar behavior? The most plausible explanation lies in the fact that non-violent States were under the full control of one group. Meanwhile, places with more than one group had the highest number of homicides.

Other types of crimes appeared in the violent places as an *emergent property*, derived of the presence of local and Federal security forces. Given that the struggles between criminal groups, as well as those against security forces required enormous amount of resources (guns, ammunitions, hitmen, bribery of politicians and police officers, etc.), some cartels expanded their criminal actions, and they started to obtain resources from extortion, payment for protection, illegal gambling, prostitution, etc.

Additionally, the largest Mexican cartels also dedicated several time and uncountable amount of resources in money laundry, providing cartels with secure and legal sources of capital. That is the reason for which key elements in drug organizations were accountant for. The most important result of this money management was the return of illegal capital to the legal market. Cases of money laundering were even allegedly performed by a US bank^8^. These transfers of capital have increased the difficulty of reducing the strength of those criminal organizations.

All the aforementioned context can give us an idea of the power reached by cartels, the importance of the control of a territory such as the MMA, and the cost in terms of lives that the Golfo-Zetas war left behind, besides the economical and social losses. In what follows, we will provide a brief resume of the main reasons that derived in the Golfo-Zetas war in the MMA.

Allegedly, the fight between the *Cartel del Golfo* and *Zetas* group, two of the most important criminal organizations in the North-eastern part of Mexico, was generated by a series of homicides of cartel leaders in both sides. In particular, the killing of a Zetas leader, Víctor Peña Mendoza, alias “El Concorde 3”, by a gunman of Cartel del Golfo, at the beginning of 2010 in the neighboring state of Tamaulipas (https://tinyurl.com/y2oogsy9^9^), triggered a wave of retaliations between both criminal gangs, unleashing an unprecedented number of homicides in Nuevo León, Tamaulipas and Veracruz states in Mexico.

The largest part of homicides during the FCH’s drug war was due to three main reasons:

1. Confrontation between rival criminal gangs,
2. execution of rivals,
3. and confrontation between criminal groups and Mexican security forces (Army, Navy, State Police).

Importantly, the last years of the homicidal time series in Fig. 2 reflect that homicidal violence in Mexico increased even more than the first rise of homicides in absolute numbers. On the other hand, Nuevo León and MMA homicides did not reach the low levels observed prior to FCH’s arrival, despite their maintenance at relatively low numbers (compared to the highest peak).

The aforementioned rise of violence during the second part of EPN’s administration, was not only due to the drug war, or confrontation between drug cartels and Mexican security forces. It has been demonstrated that drug-band related homicides have a strong influence in general homicides^10^. The increasing homicide rates have thus permeated in Nuevo León and the MMA.

## Geo-social MMA background

MMA is composed by 19 municipalities, namely, Monterrey, Guadalupe, San Nicolás de los Garza, Apodaca, San Pedro Garza García, Santa Catarina, General Escobedo, Juárez, Hidalgo, Santiago, Cadereyta Jiménez, García, Salinas Victoria, Pesquería, Ciénega de Flores, General Zuazua, Marín, Carmen, y Doctor González. The number of neighborhoods within these municipalities comes to 2691, where more than 4.5 Million people live.

Neighborhoods can tile up a city in a way where each tile is a village of its own. Studies about cities in the United States have shown that crime is highly concentrated in some neighborhoods within cities^11^. However, a typical urban tiling comprises hotspot neighborhoods bordering low crime-rate ones^12^.

It is not uncommon in Latin America to have extreme cases of slums next to luxurious neighborhoods separated typically by urban infrastructure. MMA is not the exception; while San Pedro Garza García is the second municipality in Mexico in terms of human development index (HDI = 0.901 in 2015^13^), the municipality of Santa Catarina (Northern border of San Pedro Garza García) has neighborhoods with important levels of poverty. Moreover, neighborhoods have been shown to be interdependent in terms of what happens in one affects the other^14^. In particular, crime shows diffusion processes across neighborhoods^15,16^.

## Approaches to understand spatial and temporal evolution of violence

There have been many attempts to describe, understand, predict or control the dynamics and spread of conflict and gang-related violence: from literature-based approaches^17^, data-mining-based network inference^18,19^, reaction–diffusion equations^16^, to combined methods^20,21^. Understanding temporal and spatial evolution of homicides in a metropolitan area is of utmost importance to alleviate and diminish said violence. Several other works have been done regarding spatial and temporal analysis of crime^22–30^.

For example, Caminha et al.^24^ showed that the patterns of property crime coincide more precisely with human mobility rather than fixed population. They found a linear correlation between property crimes and floating population. They suggest that property crimes occur more often in places with higher patterns of floating populations.

Ribeiro et al.^25^ unveiled relationships between crime and property in England and Wales via density scale-adjusted metrics and network tools, focused on the problem of under-representation of rural populations to scale measurements of crimes in England, in particular burglary and robbery.

The large majority of references regarding the understanding of spatio-temporal crime in cities has been done using burglary or robbery, because fortunately, there is not almost data of larger crimes such as homicide. In this sense, war-related works have been performed to understand these patterns of evolution, such as those by Lewis Fry Richardson^31^, where they found a scaling law over size of population and conflict, using data from WWII.

A crucial step in the analysis and development of accurate models of homicide-dynamics is data collection, in particular, availability of geolocated data and precise dating of events is required. In this sense, works such as the one developed by Oliveira et al.^32^, have collected crime data of robberies and burglaries.

Other approaches have used homicide data, however, the level of granularity for these data is in the best cases by municipality or provinces/states^4,33^, or by country^31^. In both cases: lack of precision in geolocated data and large time-steps limit the description of a complex phenomenon such as the spread of crime-related homicides throughout a metropolitan area.

Other examples in which a network approach was used to understand the dynamics of gang-related homicides in Chicago can be observed in^21^. There, networks were constructed using gangs as nodes and directed homicides between gangs as directed links. In that case, space was not used to determine evolution on the disputed territories.

A refinement of^21^ was developed by the same group in 2013^20^. There, an exponential random graph model was used to measure the probability that a member of a determined gang was shot/killed by a member of another gang. That probability was dependent on factors such as prior history of conflict between gangs, geographical closeness, retaliation, or reciprocity between criminal groups.

Brantingham et al.^34^, also discussed the role of geographical boundaries in a gang-related conflict. By using violent crime data from the Hollenbeck Policing Division of Los Angeles, they determined, based on a Lotka-Volterra model that the number of violent crimes decreases with distance, and also, predicted boundaries between gang territories.

Despite the huge amount of literature related to crime evolution in time and space, a scenario such as the one presented in MMA during the drug war period is not so frequent (fortunately). Newspaper *El Norte* has documented the homicide violence in the MMA from the year 2011 until February of 2018. Its database reports daily homicides with number of casualties for each event and the associated longitude and latitude coordinates. To our knowledge, this is the most accurate and comprehensive geo-located homicide database for any place in Mexico.

## Outline

In this work, we used geo-located homicide data to analyze the structure and dynamics of homicide-related violence in the MMA. Data was taken from *El Norte* newspaper daily-updated database (ENDB). We developed a spatial and temporal analysis of the homicides in the MMA.

We first analyzed the time series of homicides at municipality (city), locality, and neighborhood level. By means of neighborhood-derived analysis, we assembled a network where neighborhoods are connected if they are adjacent and if there was at least one homicide during a given time window. Said networks were intersected by periods.

Second, to detect whether or not violence was correlated in a temporal fashion, neighborhood networks were build if two places had at least one event during the same week. Couples of neighborhoods with several co-occurrences in a certain period were identified.

Finally, we correlated the places in which homicides took place with the location of high-speed roads or highways from Open Street Maps (OSM) to assess if these physical urban boundaries also act as separators between crime sectors.

In previous works, information regarding suspects and victims have been provided, however, in this case, we only have information regarding the place in which the homicide occurred. The group in which the victim participated (if any) is not available. That is the main reason for which we decided to use a 1-week-window to correlate events, and observe whether or not a relationship emerges.

The approaches used here allow us to ask different questions related to the dynamics and structure of homicidal violence: Is violence in MMA spreading through time? Is violence spreading through space? Do municipalities in the MMA present a concerted pattern of violence or is there a correlation among them? Do neighborhoods in the MMA present a concerted pattern of violence or is there a correlation among them? Is violence bursting simultaneously or a time-window appears between violence in different places? Are there high-speed roads or highways related to the location of homicides?

## Results

### Homicidal violence in Mexico was not homogeneous across the country

Figure 2 shows the monthly official data of homicides per 100,000 habitants in Mexico, Nuevo León, and the area given by the Monterrey Metropolitan Area (MMA) from 2000 until 2019. The states of Chihuahua and Yucatán are added for comparison. As it can be observed there is a steady increase in the number of homicides starting in 2007 until 2012 at national level, and a second one from 2016 to 2018. The year of the first increase in homicides (2007) coincides with the start of the drug war started by president Felipe Calderón.

Chihuahua, the most violent State in México during the FCH’s Drug war, shows an increase in homicides since 2007. This dramatical rise in drug-related homicides coincides with the launching of **“Operativo Conjunto Chihuahua”**, a FCH’s initiative to decrease the alleged violence that appeared in the previous year^5^. More than 2500 soldiers and policemen were sent to Chihuahua to diminish the rates of violence^5^.

Contrary to the expectancies, that operation increased the homicide levels to unprecedented rates. Juárez City, the border city neighbor of El Paso, Texas, became the most violent place in the world in 2007, having almost 3000 casualties, due to the battle between the criminal groups Cártel de Juárez, Cártel de Sinaloa (leaded by Joaquín “El Chapo” Guzmán), and Los Zetas group.

In the case of Nuevo León, the first steady increase in homicides happens almost 3 years later, at the end of 2009 and reaches almost 10% of all homicides in Mexico in 2011, the peak of the narco war in Monterrey. The permeation of the drug war reached MMA at the end of 2009 and directed the fluctuation of homicides at a State level. In the figure we also depict some considered key moments in the evolution of the homicide rates in Nuevo León. In the following sections we will explain more broadly these moments.

We also observe a second smaller increase in Nuevo León after 2014, posterior to a steady decrease in homicides during the period 2012–2014. Yucatán, a state with one of the lowest homicides rates in Mexico, does not present the fluctuations associated with the war on drugs.

The case of Yucatán State, is also remarkable, since the drug war did not affected that place in terms of homicide violence. With these four states, as well as with the national-level homicide rate we show how the homicide violence behaved differently during the drug war period. In what follows we will show the spatio-temporal analysis of the MMA homicide evolution.

### Homicide Evolution in MMA was not homogeneous in all municipalities

**Figure 3:**
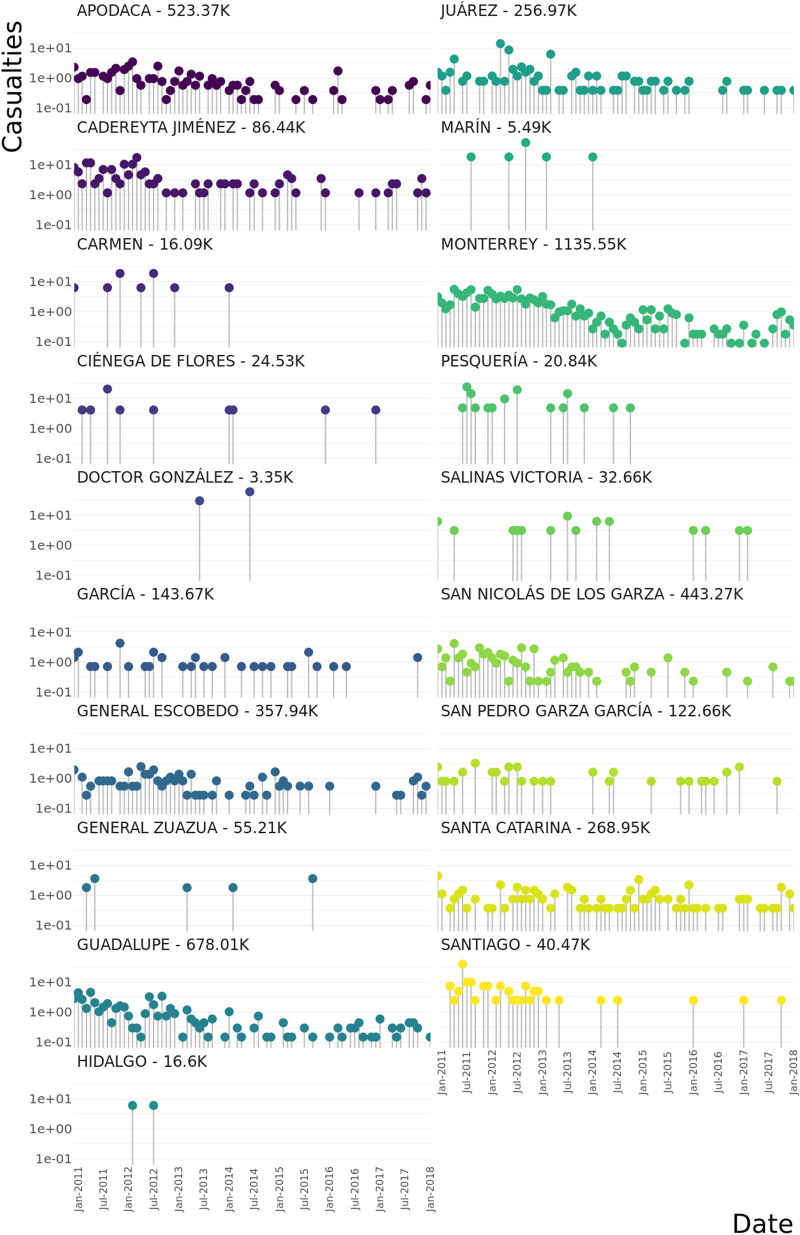
Weekly timeseries of homicides in the cities of MMA. This *lollipop plot* shows the number of casualties for each municipality of MMA during the period under study as well as the population of each municipality (next to name). The upper right panel shows the cumulative number of homicides in the “El Norte Data Base”. As it can be observed, the trend of ENDB data coincides with MMA homicides reported by INEGI in Fig. 2.

Figure 3 shows the casualties by municipality in the MMA in the form of a lollipop plot. Each point represents an aggregation of the monthly casualties per one hundred-thousand inhabitants in each municipality. There is no homogeneity in homicide rates across municipalities, revealing crime focal points located within some municipalities during this period. This is mainly the case of Apodaca, Monterrey, Cadereyta Jiménez, San Nicolás de los Garza and Guadalupe. The distribution of homicide events is denser around the 2011–2012 period, decreasing around 2014 and increasing again after 2016, coinciding with the homicide trend at the Nuevo León state level from Fig. 2.

Of particular interest is the case of Santa Catarina municipality: despite it not being one of the top places in terms of number of casualties in MMA, it consistently presents homicides during the whole period, even during the last months of 2017 and beginning of 2018.

**Figure 4:**
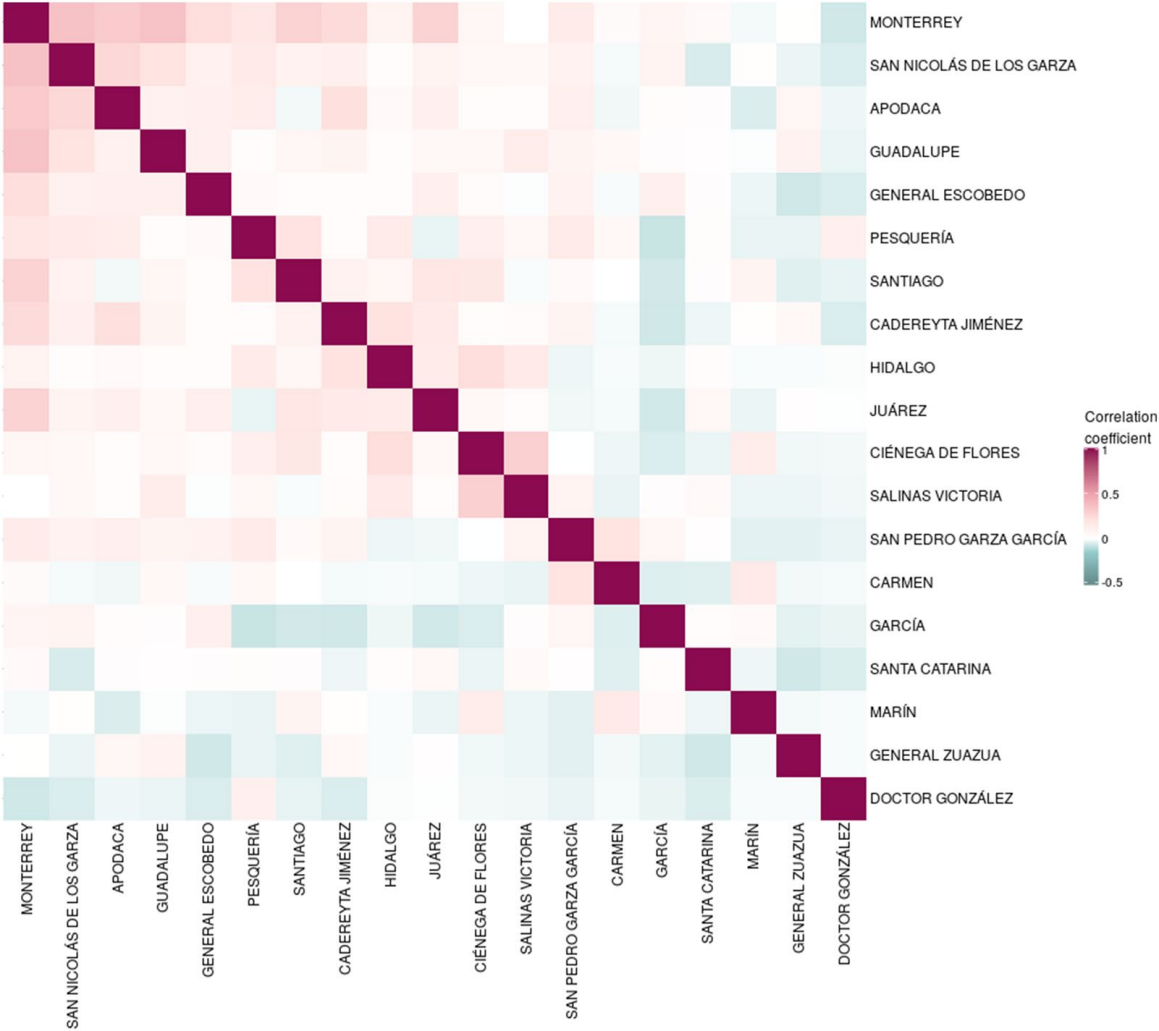
Correlation of homicides in the cities of MMA. This heatmap represents the Pearson correlation coefficient of the homicide time series of the 19 municipalities that compose the MMA. Pink squares show positive correlation, meanwhile negative correlations are depicted in blue.

The homicide time-series were used to correlate violence across municipalities across the 86-month period on a weekly basis (Fig. 4). The highest correlated city-pair is Monterrey and San Nicolás de los Garza, meanwhile García and Pesquería are the highest anti-correlated municipalities. We assigned a significance score via Z-scores to the correlation values by means of a null-model built from reshuffled iterations of the casualties by municipality (Supplementary File 1). Significantly correlated (Z-score $> 3$) municipality pairs are displayed in Table 1.

Based exclusively in the correlations observed in Fig. 4, where the most correlated cities (Monterrey and San Nicolás de los Garza) are adjoined, and the most uncorrelated places (Pesquería and García) are very distant, it is plausibly to assume that homicide violence depends on distance between places, similar to those reports from Alves et al.^23^. However, as we will show in the following sections, the detail of homicide evolution is lost using this coarse-grained approach.

### Spatial distribution of homicides

**Figure 5:**
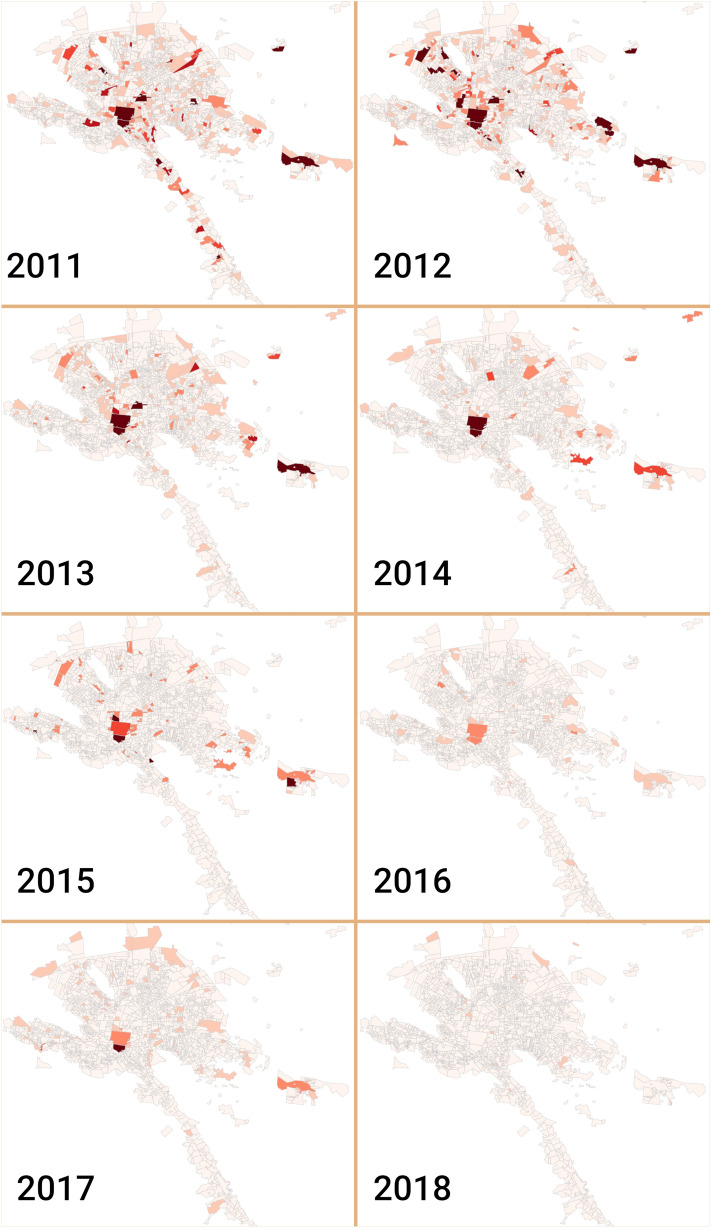
Geospatial yearly distribution of homicides in MMA. In these maps, neighborhoods are depicted according to the number of homicides that took place there. Light colors represent lower number of casualties, meanwhile dark colors take account for higher homicide numbers.

In order to have a more accurate description of the dynamics of homicidal violence, we observed the neighborhoods in which events occurred. In Fig. 5, we depict the number of casualties per neighborhood for all years under study. As it can be appreciated, the patterns of violence change over time and space periods. Importantly, we can observe the above-mentioned decrease in homicides after 2013.

**Table 1:** Significant Pearson correlation coefficient and Z-score of weekly aggregated casualties at the municipality level.

Municipality 1	Municipality 2	PCC	Z-score
Monterrey	San Nicolás de los garza	0.3581	6.4388
Guadalupe	Monterrey	0.3557	6.3556
Apodaca	Monterrey	0.317	5.6961
Ciénega de flores	Salinas victoria	0.2893	5.4055
Monterrey	Santiago	0.2839	5.0035
Juárez	Monterrey	0.2788	4.8732
Apodaca	San nicolás de los garza	0.2547	4.6693
Cadereyta jiménez	Monterrey	0.2422	4.2779
Ciénega de flores	Hidalgo	0.2263	4.143
General escobedo	Monterrey	0.2291	4.0746
Cadereyta Jiménez	Hidalgo	0.2053	3.8278
Apodaca	Cadereyta Jiménez	0.2149	3.8185
Pesquería	Santiago	0.2027	3.7373
Guadalupe	San Nicolás de Los Garza	0.2014	3.6547
Carmen	San Pedro Garza García	0.1992	3.4842
Juárez	Santiago	0.1829	3.1747
Monterrey	Pesquería	0.1756	3.0951
Ciénega de Flores	Santiago	0.1714	3.0139

**Figure 6:**
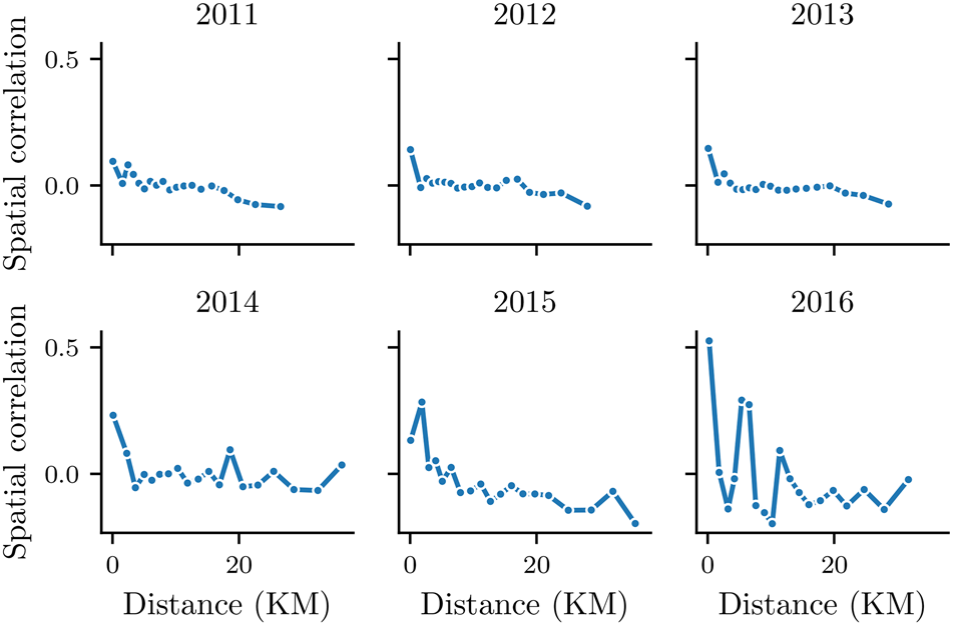
Evolution of the spatial correlation of homicides across neighborhoods and years.

### Spatial evolution of homicides

In order to better understand the change in homicide patterns across time, we use a finer level of granularity of MMA: neighborhoods. We counted a total of 2699 neighborhoods in 19 municipalities, where 769 of these neighborhoods have at least one homicide in the January 2011–February 2018 time-window. In other words 28% of all neighborhoods in MMA suffered a homicide within their boundaries. This contrasts with the known property of focalized crime on a small number of neighborhoods within a city^11^.

To analyze the dependency of distance between any two neighborhoods in the database, we calculated the spatial correlation as reported in^23^. There, number of homicides in neighborhoods were used to correlate all couples of neighbors and determine the dependency of the distance between both places. Figure 6 shows the spatial correlation of all couples of neighborhoods depending on the distance between them.

As it can be appreciated from the figure, there is no dependency on the distance between neighborhoods in the first years. Correlations are close to 0 in all ranges of distance. However, for 2014–2016 period, a small amount of correlation appears between close neighborhoods.

To look at the evolution of homicides across space, we constructed a homicide network representing the spatial configuration of violence within a given year, for each year in our time-window. In the homicide network nodes are neighborhoods and two nodes are connected by an edge if there was at least one homicide in the two neighborhoods in the same week. Edge weights are assigned from co-occurrences between same neighborhoods. Table 2 shows neighborhoods with a high number of co-occurrences within a same week.

**Table 2:** Neighborhood pairs with more than three casualty co-occurrences in the same week for a given year.

Co-occurrences	Neighborhood	Neighborhood	Year
12	Cadereyta Jimenez Centro	Centro de Monterrey	2012
10	Centro de Monterrey	Independencia	2012
8	Cadereyta Jimenez Centro	Independencia	2012
7	Cadereyta Jimenez Centro	Centro de Monterrey	2011
6	Cadereyta Jimenez Centro	Benito Juarez Centro	2012
6	Benito Juarez Centro	Centro de Monterrey	2012
5	Centro de Monterrey	Moderna	2011
5	Cadereyta Jimenez Centro	Arturo b de la Garza	2011
5	Cadereyta Jimenez Centro	Riberas de la Silla (Fomerrey 31)	2011
5	Centro de Monterrey	Francisco Villa	2012
4	Francisco Villa	Pesqueria	2011
4	Centro de Monterrey	Francisco Villa	2011
4	Cadereyta Jimenez Centro	Moderna	2011
4	Centro de Monterrey	Concepcion Salazar	2011
4	Francisco Villa	Moderna	2011
4	Centro de Monterrey	Arturo b de la Garza	2011
4	Benito Juarez Centro	Arturo b de la Garza	2012
4	Cadereyta Jimenez Centro	Plutarco Elias Calles	2012
4	Cadereyta Jimenez Centro	Francisco Villa	2012
4	Centro de Monterrey	San Bernabe Fomerrey 25	2012
4	Cadereyta Jimenez Centro	La Campana	2012
4	Cadereyta Jimenez Centro	Mitras Centro	2012
4	Benito Juarez Centro	Independencia	2012
4	Centro de Monterrey	Mitras Centro	2012
4	Independencia	La Campana	2012
4	La Alianza	Centro de Monterrey	2012
4	Centro de Monterrey	La Campana	2012
4	Cadereyta Jimenez Centro	Arturo b de la Garza	2012
4	Centro de Monterrey	Pesqueria	2012

**Figure 7:**
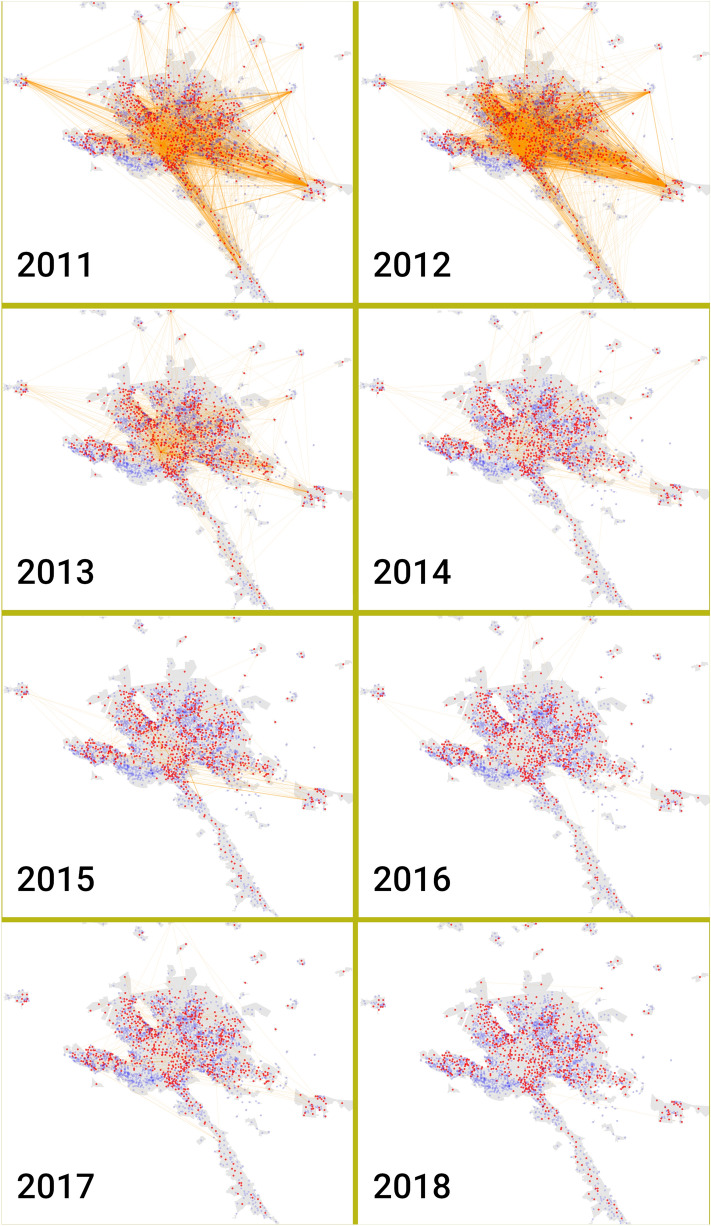
Network of homicides in MMA during the eighth years. In this network representation, red dots represent neighborhoods with at least one homicide during the measured year. Blue points are neighborhoods without homicides during the period. Links join those neighborhoods that have at least one homicide in the same week.

The analysis of each yearly crime network shows the spatial dynamics of neighborhoods in terms of homicide (Fig. 7). The global spread of violence in distant focal points is seen across all years (scattered red points, neighborhoods with at least one homicide that year), suggesting a division in crime sectors (blue points are neighborhoods without homicides).

The structure of crime sectors however, varies greatly between years. In years 2011 and 2012, there are multiple edges connecting nodes from outer violent neighborhoods, such as Cadereyta Centro to Monterrey Centro neighborhoods. The number of edges decreased steadily until 2014 onwards.

**Figure 8:**
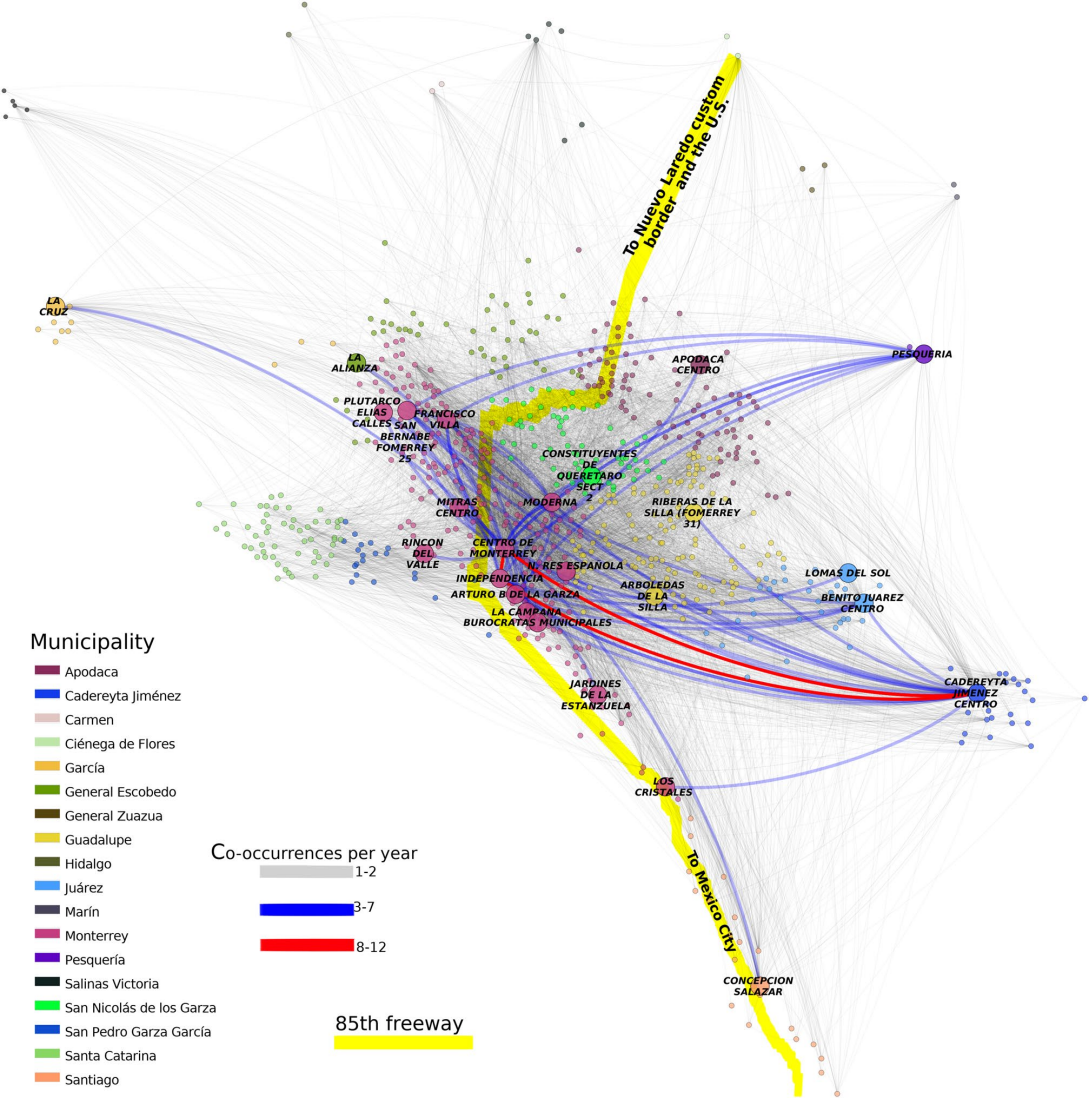
1-week correlation network. This network shows those neighborhoods with at least one correlation, given by the appearance of at least one homicide in the same week. Neighborhoods with homicides but without correlation are not depicted. Each color represents the municipality of the neighborhood. Grey links represent couples of neighborhoods with one or two co-occurrences. Blue links join neighborhoods with 3-to-7 co-occurrences during 1 year, and the red ones shows those neighborhoods with 8–12 co-occurrences in 1 year. Big circles show neighborhoods with red/blue links. It is also depicted the route of 85th freeway, which cross the entire MMA and communicates this state with the US custom border in Nuevo Laredo City.

To have a more accurate description of the homicidal evolution in space, we used the same network from Fig. 7, highlighting those links with more co-occurrences in a certain period. Thus, in Fig. 8 we depict the neighborhoods with at least one weekly correlation. Neighborhoods are colored according to their municipality. Link color depends on the frequency of co-occurrences between the same couple. Red links represent those places with 8 or more co-occurrences in 1 year. Blue interactions take account of couples with 3–7 co-occurrences in 1 year, and grey thin lines join neighborhoods with one or two co-occurrences during a given year. Finally, a yellow line representing the 85th freeway is also depicted.

In the figure it can be appreciated that the more frequent links occur between periphery neighborhoods and the Downtown zone of Monterrey City. It is possible to depict a polygon that contains the Monterrey’s Downtown (Centro de Monterrey), Moderna, Independencia, Arturo B de la Garza, Nueva Res Española, La Campana and Burócratas Municipales neighborhoods.

The large majority of blue and red links join neighborhoods of the peripheral municipalities with the aforementioned polygon. Additionally, almost all the blue/red links join neighborhoods from Monterrey City and any other municipality. Furthermore, there are not abundant weekly co-occurrences between close neighborhoods.

The star-like structure of the network observed in Fig. 8, reflects two interesting properties: (a) The most important correlations in terms of co-occurrences appear between distant places and, (b) Several neighborhoods with high correlations are crossed by the 85th freeway. The set of co-occurrences during the whole period is reported in Supplementary File 2.

### Social barriers in the homicide evolution

**Figure 9:**
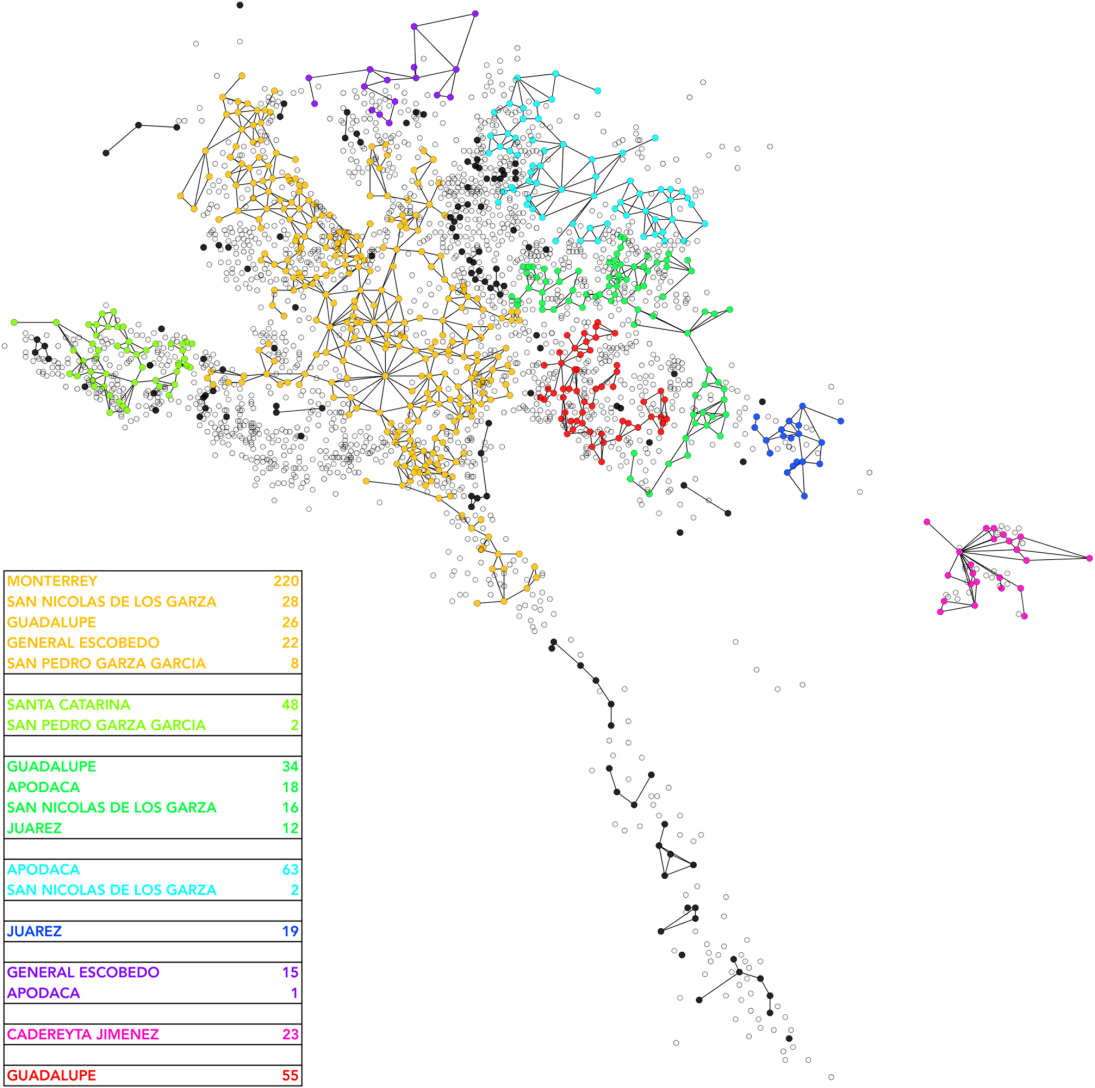
Network composed by adjacent violent neighborhoods during the entire period under study. Each color represents a connected component with more than 10 edges. The bottom left table indicates the municipalities that belong to each component. Black dots are neighborhoods with homicides but not connected to large components.

In order to have a global view of the homicidal crime, we constructed a network using the whole-period window. There, nodes are connected if they are adjacent and have at least one homicide during the 86 months. Figure 9 represents such a network, where nodes are colored by connected components with more than 10 edges (main connected components), white nodes are neighborhoods without crime (not in crime network), and black nodes belong to connected components with less than 10 edges.

It is important to remark that using the whole period to construct a network such as this last, we eliminated the temporal correlation, and thus we only may observe the spatial correlation. Being more precise in the description, with this network we try to observe the lack of *correlation* between places. Since a high percentage of the MMA neighborhoods had homicide events, the size of network clusters may indicate those barriers of violence that blocked the spread of homicides.

Looking at the composition of the nodes, each one of the eight main connected components adheres to a main municipality strongly. For instance, yellow nodes belong to a main component with principal municipality Monterrey, containing 72% of the nodes. Red connected component has all its nodes in the municipality of Guadalupe, to the east. Green component in the west has 55 of its nodes in Santa Catarina.

It is worth noticing cases such as the border between Santa Catarina and San Pedro Garza García municipalities (Green and white nodes at the west). Despite the fact that several neighborhoods of both cities share a border, none of these share an edge. Interestingly, safe neighborhoods seem to be located around the municipality boundaries thus disconnecting the main connected components.

### Homicides were related to urban environment during 2011–2012 period

As we saw in the previous result, crime connected components or crime sectors are usually located within municipalities. Although interesting by itself, the fact that municipality borders can act as a separator between crime sectors leads to ask whether there are other barriers to crime in the MMA.

The relevance of high-speed roads or highways in crime has been previously shown to be an important factor^35^. Specifically in the context of a time window comprising part of a drug war, highways are of high relevance: they are usually the spots where bodies were abandoned or displayed, persecutions between criminals and police/military take place on highways, etc. Moreover, highways crossing a city act as an urban boundary. They separate neighborhoods, and municipalities, and can separate social and urban landscapes.

In order to include highways in our data, specifically in order to observe the proximity of crime events near a highway and also analyze the distance of violent neighborhoods from them, we obtained all Open Street Map (OSM) data points related to highways in the MMA.

**Figure 10:**
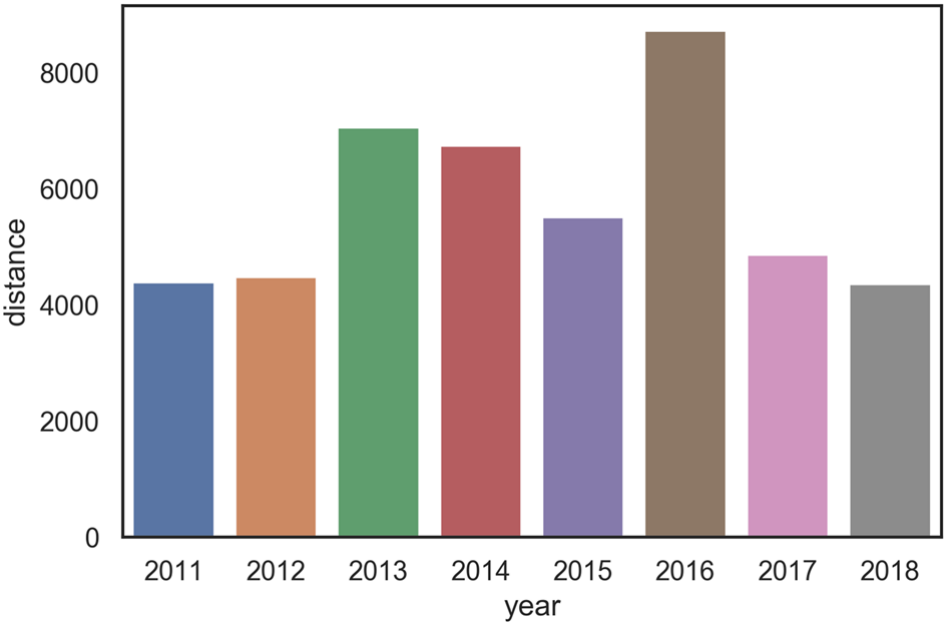
99th percentile in meters of distance from highway to crime by year.

As we see in Fig. 10, distance from crime to highway varies between years. In order to ignore outlier points we consider the 99th percentile (distance such that 99% of crimes are closer than or equal to a highway) over the 8 years. The 2 years related to the climax of the war on drugs (2011–2012) show the shortest distances to highways (4379 and 4475 meters, respectively). The time period between 2013–2016 shows longer distances to highways, and a decrease in distance is observed again in 2017.

**Figure 11:**
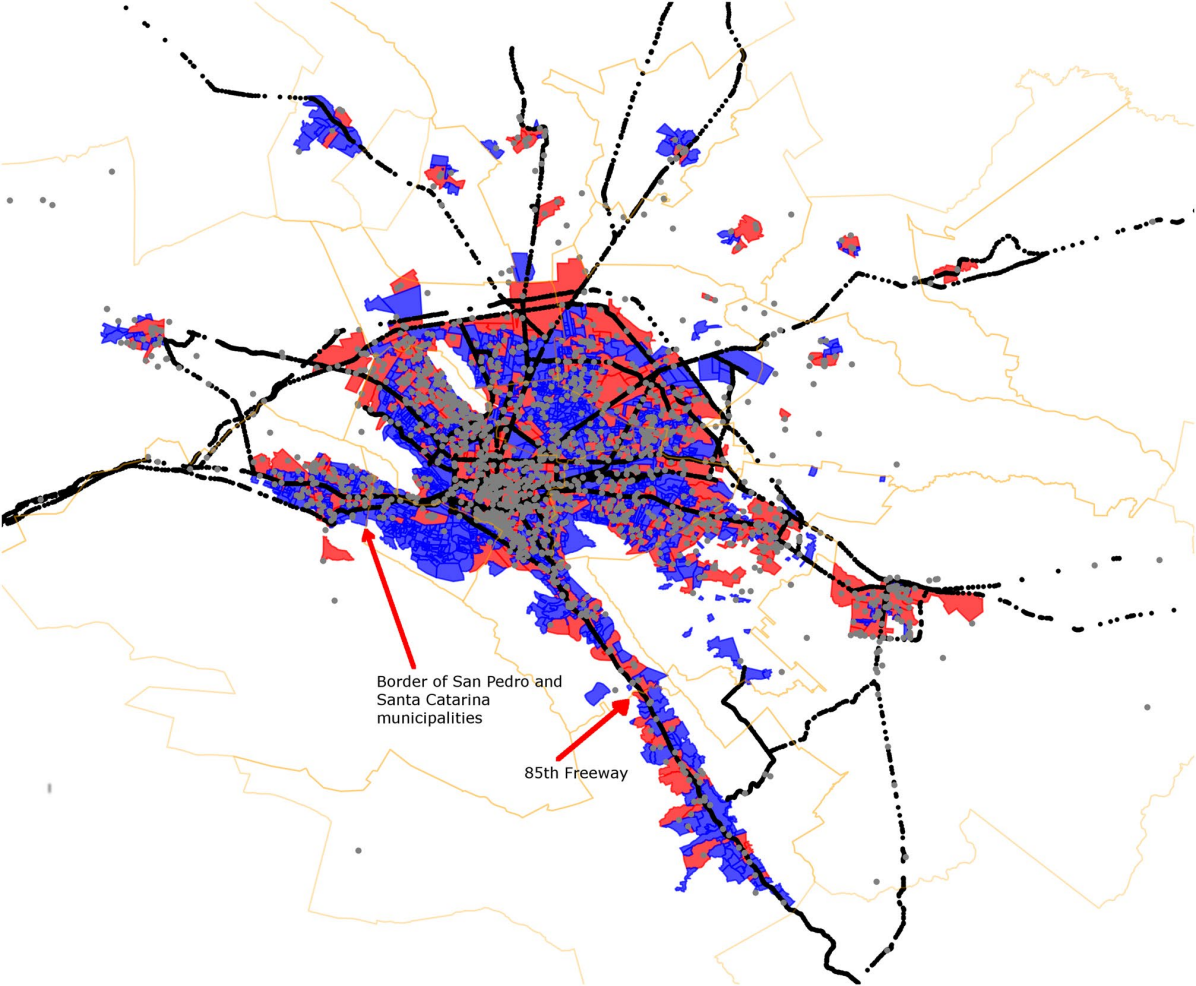
Geospatial distribution of homicides in MMA with highways.

In general, highways appea to be part of a backbone of violence as many of the homicides lay directly on the highway paths (Fig. 11). It can also be appreciated how the political division together with the highways could be forming the patterns between violent and non-violent neighborhoods (red and blue neighborhoods, respectively). Indeed, the polygons resulting from the intersection between municipalities and highways appears to unveil crime patterns and should be further investigated. One of the most evident effects of this separation is again San Pedro Garza García and Santa Catarina municipalities.

As an additional note, in Fig. 9 we observe the 85th freeway. This path connects the center of the country with the US border (the freeway starts in the Center of Mexico City, in the Presidential Palace and ends in the US custom border of Nuevo Laredo, Tamaulipas). In the figure it can be appreciated that the freeway cross 11 out of the 25 highly correlated neighborhoods (circles with names). Importantly, the 85th freeway crosses the aforementioned polygon in the center of Monterrey City; furthermore, it also connect the Santiago municipality with Monterrey City.

## Discussion

In this work, using a geolocated homicide database of Monterrey Metropolitan Area (MMA) during the period 2011–2018, we have implemented a network approach to describe the spatial and temporal evolution of the homicidal violence in this important economical and social region in Mexico.

By means of these well-curated data we have approached to a phenomenon of drug-war-related violence with no precedents in Mexico. This is the first time, to the best of our knowledge, that a network of geolocated homicides is constructed for a place affected by a drug war.

We constructed networks to dissect the temporal and spatial dynamics of this violence. As a summary of methods, in this work we:

- Analyzed the time series of homicides in different cities of the MMA, to observe whether or not a global correlation appeared.
- Analyzed the spatial correlation between places where homicides took place, to observe whether close distances influences the frequency of homicides.
- Constructed yearly co-occurrence networks, by correlating neighborhoods with homicides in the same week, and counting the co-occurrences of these couples in 1 year.
- Constructed a network by aggregating all data of homicides, eliminating the temporal correlation, in order to observe whether homicide clusters appeared, and how those clusters looked like.
- Correlated the location and frequency of homicides with roads, freeways and highways, to observe whether a trend on the homicidal violence appeared.

These approaches have not been used in data such as the one presented here. In what follows, we will try to put into a broader context the findings obtained here, in an attempt to explain the reasons for which that violence emerged and continued during several years in MMA.

### Temporal evolution of the homicidal violence

From Fig. 2 we observed how the homicide violence in Mexico was so different depending on the state under study. Hence, being the violence in Mexico triggered by the former president Felipe Calderón (FCH) drug war, the dramatical differences between states was clearly due to specific and bloody battles between groups to control certain territory. Those groups could be part of the organized crime, or also composed of Mexican security forces (army, navy, federal, state or local police). Some places in Mexico, such as the State of Yucatán were not importantly affected.

Focused on the MMA, where the geolocated homicide data is obtained, we observe a series of results that call our attention: In the majority of municipalities, an important increase in homicides is shown in the period 2011–2013, decreasing until 2016, and with a small increment in the last months of 2018, but only in some places, such as Apodaca, Cadereyta Jiménez or Monterrey.

At the same time, Santa Catarina Municipality shows a barely constant homicide dynamics, being the only one with this behavior. This is also reflected in the fact that this municipality presents the lowest correlation values with aggregated data (Pearson Correlation matrix of Fig. 4).

Additionally, the highest correlation coefficients contains close municipalities: Monterrey, San Nicolás de los Garza, Apodaca and Guadalupe (Fig. 1). This is in agreement with other reports in which the closest distances correlate with the dynamics of homicides^23^. However, by using the same approach that the one reported in^23^, Fig. 1 shows clearly that there is no spatial correlations in the homicide violence in MMA, at least during the period 2011–2016. This was the reason for which we decided to analyze other forms of correlation, trying to understand the dynamics of violence in MMA.

The correlation matrix of Fig. 4 was constructed using the 8-year period. This long period could destruct short-term spatial correlations; additionally, the municipality is a coarse grained description, because the data used for this work was much more accurate (longitude and latitude). That is why we decided to construct a network on the neighborhood level.

As Fig. 4 shows, the yearly evolution of homicides in MMA varied importantly between the same municipality. To overcome the loose of temporal detail, we decided to use a 1-year time window to analyze the temporal correlation between neighborhoods. We constructed a co-occurrences network where two neighborhoods were joined together if they had homicides in the same week. The higher the number of co-occurrences in 1 year, the strongest the correlation between these neighborhoods.

2011, 2012, and 2013 were the years with the highest rates of homicides, also showed the highest co-occurrences. A reasonable question is how the violence disseminate over all the MMA and more importantly why this occurred? A possible explanation could be behind the results observed in the Fig. 8.

The weekly co-occurrence network observed there, shows only 25 out of the 700 neighborhoods in MMA with 3 or more weekly co-occurrences in the whole period under study. The majority of links with more than 3 co-occurrences (blue and red links), appear between peripheral neighborhoods of external municipalities and correlate with the Downtown Zone, which contains 9 Monterrey City neighborhoods.

These places form a polygon in which the large majority of weekly co-occurrences took place. Several homicides in the polygon occur, as well as the respective retaliations in the periphery. This starlike behavior allows us to suggest that the overall landscape of homicide violence occurred for the control of the Center of Monterrey City.

Another point to take into account that also reinforces the hypothesis of the Polygon control, is that there is almost none co-occurrence between adjacent or close neighborhoods in any part of the MMA during the study, but those in the Polygon zone. This is evident from the observation of Pesquería and Cadereyta municipalities and their neighborhoods, practically all of them had co-occurrences with other municipalities, mainly in the Polygon zone.

It is worth noticing that we do not know the direction of those links. Since information regarding the offender and the victim is lacking, we only count with the location and date of occurrence.

Interestingly, the Polygon is crossed by the 85th freeway, also known as “Carretera Panamericana”. This highway starts in the Presidential Palace in Mexico City, ending in the U.S. Custom border of Nuevo Laredo, in the State of Tamaulipas. Nuevo Laredo Custom Border is the most important US-Mexico trading place (USD$180,000,000,000.00 last year^36^). This is the most direct highway to reach that border.

A complementary hypothesis is that the huge number of homicides in MMA was due to control not only the Polygon zone, but more importantly, for the control of the 85th freeway, and with that, the transport of any kind of merchandise.This hypothesis is reinforced by the number of casualties observed in results observed in Fig. 11 close to the 85th freeway. It crosses the Santiago municipality, where several homicides took place, and also cuts the polygon zone.

To note that the 85th freeway is not the road with the highest levels of transit. The annual average daily transit (TDPA) for this road is 17,500 cars. The one with the largest average traffic during a year is the 40D highway. This road connects the Saltillo City (Capital of Coahuila State) with Monterrey City. Its TDPA is 37,200, more than twice the traffic reported in 85th freeway^37^.

The most frequent co-occurrence was Monterrrey downtown with Cadereyta Downtown; this is, another city in the Southern East side of the MMA. However, Cadereyta Centro was also correlated with several other polygon neighborhoods. This phenomenon was observed not only in 2011–2012 period, but also in further years, as shown in Fig. 9.

### 85th freeway and the control of MMA

We provide an explanation for which this freeway is the most correlated road to homicides: As we previously mentioned in this response, as well as in the manuscript, the increasing of homicidal violence in MMA and Nuevo León State in general, was originated by a struggle between El Cartel del Golfo and Los Zetas. This last group was an excision of the first one. Zetas was the armed force of Cartel del Golfo. The name Cartel del Golfo was adopted because this group controlled the states of the Eastern Coast of Mexico: Tamaulipas and Veracruz. Additionally, this cartel controlled Nuevo Leon, Part of State of Coahuila, Zacatecas and San Luis Potosí. However, the most important points of control was Tamaulipas, Nuevo León and Veracruz.

In Fig. 8 we also show the places in which these events appear. It is interesting that the neighborhood with more co-occurrences in the MMA was Monterrey Centro; however, the second most connected one was Cadereyta Centro. And more importantly, Cadereyta Centro neighborhood had clearly more (and more frequent) co-occurrences with long-distance places. That is observed by the blue and red links appearing in that place.

This is another instance in which this network approach helped us to describe the spatio-temporal evolution in MMA, and also gave us hints to provide possible explanations for which the violence behaved like that.

### Socio-economic segregation of homicide violence

Regarding the temporal evolution of crime, an interesting result came from data of Santa Catarina municipality. As mentioned in the results section, Santa Catarina is not the most violent municipality in MMA, but this place has a monotonous homicide rate, even during the years of low homicide rates in MMA. This behavior contrasts with the rest of municipalities of the MMA: It can indeed be appreciated in Fig. 4, where Santa Catarina’s correlations are the lowest for the entire set.

On the other hand, the other municipalities with high homicide rates have a similar behavior, with an important increase in 2011–2012, short after decreasing until 2017, where violence raised again, but at lower levels.

One may argue that Santa Catarina municipality had a violence evolution more or less independent on the rest of the MMA. This could be due to the fact that it shares the largest border with San Pedro Garza García municipality, the safest place in MMA, as well as the one with the highest development index per capita (0.91). In this case, the socioeconomical barrier between San Pedro and Santa Catarina, separated the dynamics of violence of the entire MMA with Santa Catarina municipality. In this sense, San Pedro Garza García served as a homicide buffer of Santa Catarina; this broke the *synchronization* with homicide dynamics in the rest of MMA.

Opposite to Santa Catarina, San Pedro Garza García municipality has a very low homicide rate (150 and 44 homicides, respectively). These two cities are separated by one avenue: Cromo Street. In the side of San Pedro there were no homicides, as opposite to Santa Catarina, where several neighborhoods suffered from homicidal violence. This contrast can be attributed to the difference in the GDP between both places. San Pedro Garza García is the second city with the highest GDP in Mexico^13^, meanwhile some neighborhoods in Santa Catarina have important development lag. According to the last economical survey (2015), the GDP *per capita* in San Pedro Garza García was $25,636 USD, meanwhile that of Santa Catarina was $10,783 USD.

Similar to this behavior related to revenue per capita, other municipalities such as San Nicolás de los Garza, have a small number of neighborhoods with homicides compared to the rest of municipalities within the city. However, in the case of San Nicolás de los Garza, homicides occurred chiefly in its periphery. This can be observed from Fig. 11, several neighborhoods on its central area are in blue, meanwhile the borders are in red (north-east of MMA).

Concomitant with the political borders of municipalities, highways also appear to depict lines of segregation between secure and violent neighborhoods. The remarkable difference between neighborhoods at opposite sides of a highway may reflect the presence of security forces in only one of both sides. Further investigation regarding this topic is needed to reach a solid conclusion, however, we discuss here the empirical observation.

### Networks and geopolitical division of homicide

The crime network constructed from joining adjacent neighborhoods if they had at least one homicide during the time window, clearly shows that homicide events are linked to the municipality in which the homicide occurred. Similar to the aforementioned relationship between GDP and violence, here we observe that homicide regions are strongly segregated by the political division of the MMA: network components belong to practically just one municipality.

This last result is reinforced with Fig. 9, where each component belongs to one municipality with a high proportion (Table 3). In this sense, a very interesting case is the one corresponding to San Nicolás de los Garza and Guadalupe municipalities, as they share neighborhoods with other violent municipalities in the crime network. On the one hand, Guadalupe has two main connected components associated with it: a cluster exclusively composed by Guadalupe’s neighborhoods, and another with neighborhoods shared with other two municipalities, i.e. Monterrey, San Nicolas, Apodaca and Juarez. Finally, there is no main connected component in San Nicolás de los Garza holding a substantial number of neighborhoods.

Probably, both phenomena should be explained by the fact that they are transit-only places. The hypothesis may apply better to the case of San Nicol´as, as it does not have a main connected component.

**Table 3:** Proportion of main municipality within a main connected component.

Connected component	Number of neighborhoods	Main municipality	Proportion	Ratio
1	305	Monterrey	220	0.72
2	80	Guadalupe	34	0.42
3	65	Apodaca	65	1
4	55	Guadalupe	55	1
5	50	Santa Catarina	48	0.96
6	23	Cadereyta	23	1
7	19	Juárez	19	1
8	16	General Escobedo	15	0.94

The other fact that emerges from the crime network, is that several neighborhoods are in the middle of clearly violent areas but they do not have any casualty during the 8 years of data. As an example of this, we point to those neighborhoods between the green, yellow and red clusters in Fig. 9: All these neighborhoods belong to Guadalupe municipality. The gray nodes inside the yellow component, which belong to Monterrey, are also remarkable, but the most dense set of neighborhoods with no main connected component is the one between the yellow and cyan clusters. This set is part of San Nicolás and despite the fact that (1) this area is surrounded by three crime connected components, and (2) it also contains non-clustered homicide neighborhoods (black dots), hundreds of neighborhoods are not touched by the homicidal violence.

This last observation coincides with the hypothesis that San Nicolás de los Garza municipality was not a disputed territory, but instead a transit one. This fact is fully inline with the urban environment and its relation with homicides in MMA.

### Urban environment and homicidal behavior

By observing Fig. 11, we may corroborate that the urban environment also shapes the broader distribution of homicides in MMA during the measured period. The central polygon of Monterrey’s Downtown encompasses the most violent area of MMA during the whole period (Centro de Monterrey neighborhood).

On the other hand, we also may observe that Santa Catarina and San Pedro municipalities, despite they are together, the separation between them by one avenue, clearly distinguish the violence located in Santa Catarina, and the almost absent homicidal violence in San Pedro. As previously mentioned, it is possible to observe how roads separate places with or without homicides: Apparently the combined political, socio-economical and transportation factors may separate relatively large areas of homicidal violence.

## Concluding remarks

In this work, by means of a network approach, using a unique and carefully curated database of geolocated homicides during a period of 8 years, together with map data from Open Street Maps, we have been able to describe the spatiotemporal dynamics of homicidal violence in one of the most important urban areas in Mexico, the Monterrey Metropolitan Area (MMA).

A relevant question is why this violence decreased after those years? A possible explanation can be found in the local actions that Nuevo León Government took to face this problem.

In 2013, the former Nuevo León Governor, Rodrigo Medina, launched the *Fuerza Civil de Nuevo León*^38^, a State security force with special training and external certification, absent features in the local security forces at that moment. For the year 2014, homicides reduced in a 75% compared with 2011.

Additionally, during 2011 and 2012 several leaders of both Golfo and Zetas groups were captured or killed. However, the violence levels never reached the rates before the FCH’s drug war.

To our knowledge, this is the first time that such an amount of manually curated data is used to construct spatial and temporal networks, to provide insights of how violence increases and decreases as relative to the underlying different social turmoil in Mexico during this time window.

We have been capable to study at different levels of granularity of temporal (8-year, year, and month), and spatial components (country-level, state-level, municipality, and neighborhood), in order to provide a multi-scale approach that allows to dissect possible explanations behind the violent behavior in urban metropolitan areas, specifically in the case of the war on drugs in Mexico.

May be the most important take-home message is that a network approach such as the one presented here, have allowed us to develop data-driven hypotheses regarding the homicidal evolution in the Monterrey Metropolitan Area, providing explanations of why particular zones become the scenario of one of the most cruel battles for a portion of land and the transport of merchandise.

Further steps in this regard may focus in performing null models to corroborate whether the geo-socio-political division segregates homicidal regions in MMA. A large effort of several groups should also be made on the data collection and curation.

## Methods

### Data acquisition

Crime related homicides records for MMA were acquired from EL Norte Mapa del Crimen (2011–2018) (https://gruporeforma.elnorte.com/libre/offlines/mty/mapas/mapadelcrimen2011.htm). Web data extraction was performed for every available year to assemble a single data base: El Norte Data Base (ENDB), with the following variables: date, latitude, longitude, casualties, title of the newspaper entry reporting the event and associated URL. The total number of observations in the ENDB is 2264.

Shapefiles containing neighborhood data for Mexico were downloaded from datamx (http://datamx.io/dataset/colonias-mexico/resource/7b5a3b0a-4405-48d6-a4eb-d9f13bb50d3a). The set was filtered to keep only neighborhoods from the Nuevo León. The data set consists of 2691 polygon features with neighborhood names, municipality and geospatial data such as area and location. The official number of homicides for Mexico, the state of Nuevo León and the Monterrey Metropolitan Area (MMA) in the 2000–2018 period was acquired from the National Institute of Statistics and Geography (https://www.inegi.org.mx/sistemas/olap/proyectos/bd/continuas/mortalidad/defuncioneshom.asp?s=est).

### Events mapping

Entries from the ENDB were mapped into the shapefiles using latitude and longitude coordinates to locate the neighborhood and municipality where each event took place. Entries were kept if they were located inside a neighborhood to filter for urban areas. Mapping was performed using pandas and geopandas packages in Python. After the filter, 2114 entries in the ENDB remained.

### Neighborhood networks

Adjacent polygons for each neighborhood were determined using geopandas. Each node represents a neighborhood and nodes were linked if both nodes had an event during a determined time window in the ENDB and if they were adjacent polygons in the geospatial data. A network was build using the entire period and yearly networks were also generated. For the spatial evolution of homicides, networks were assembled by counting the number of events that were registered in the same week for two adjacent neighborhoods and by using a 1-week shifted window. Networks were created using networkx and visualization and analysis of their structural features were performed using Cytoscape.

### Municipalities correlation

The entire dataset of causalities aggregated by week was used to calculate the Pearson Correlation Coefficient (PCC) between each pair of municipalities in the MMA. A null-distribution was obtained from a thousand iterations of PCC calculation of the reshuffled causalities by week for each municipality. A z-score was assigned to each correlation value by placing the observed PCC in the null-distribution. Correlation heatmap was generated using the ComplexHeatmap package in R.

### Spatial correlation

Given a year *t* and a distance interval *r* in kilometers, the spatial correlation *G*(*t*, *r*) for all pairs $(i, j) \in N$, where *N* is the set of all neighborhood pairs within distance *r*, is computed using the formula from Alves et al.^23^, which is as follows:

$$\begin{aligned} G(t,r) = \sum _{\begin{array}{c} (i, j) \in N \\ i \ne j \end{array}} \frac{(h_i - \mu ) (h_j - \mu )}{|N| \sigma ^2}, \end{aligned}$$

where $h_k$ is the number of homicides per capita of neighborhood *k* at time *t*, $\mu$ and $\sigma ^2$ are the mean and variance of homicides per capita at time *t* and |*N*| is the number of neighborhood pairs at within range *r*.

As no data is available for the population of each neighborhood *i* of municipality *m*, we computed a proxy $A_i P_m$, where $A_i$ is the area of neighborhood *i* and $P_m$ the population of municipality *m*. This proxy was used to obtain the homicides per capita of each neighborhood. Furthermore, the distance intervals where defined by a sequence of quantile values from 0 to 1 with a 0.05 quantile step. So the first range consists of the distance in KM from 0 to the bottom 5% distance; the second from the bottom 5% distance to the bottom 10% distance, and so forth. In total there are 20 distance intervals.

### Open street map

Data from Open Street Map (OSM) was obtained using the Overpass API for the Monterrey Metropolitan Area (bbox = $(-100.8421, 25.3217), (-99.5650, 26.0346)$). Nodes and ways were parsed from the data using the pyosmium library, where ways were fined-grained to retrieve only their nodes coordinates.

## Supplementary Information

Supplementary Information.

Supplementary Infromation 2.

## Data Availability

The datasets generated and/or analysed during the current study are available in the crimenNL repository https://github.com/ddiannae/crimenNL.
